# Minutes of the Fifth Annual Meeting of the Ohio Dental College Association

**Published:** 1857-03

**Authors:** Charles Bonsall


### MINUTES OF THE FIFTH ANNUAL MEETING OF THE OHIO DENTAL COLLEGE ASSOCIATION.

The association met according to adjournment in the College building at 10 A. M. of Monday, 23rd of February, 1857.

Present: Drs. James Taylor, H. R. Smith, Chapman, Watt and Bonsall.

The following report and suggestions were offered by Dr. Taylor. President of the association for its consideration.

"Gentlemen of the Ohio Dental College Asociation: This is our fifth annual meeting and the circumstances which surround us are so different from those which ushered in our organization that a passing notice may be of some practical moment.

On Feb. 19th 1852, at 10, A. M. the stockholders of this institution met in the College Hall, and took the initiatory steps for the organization of the association which was consummated the same evening.

The association can point to this edifice as a part of *its own* work, and we think may with just feelings of pride say that no other Dental society can show such evidence of zeal in laying broad the foundations of Dental science.

This work has been done with, at no time any considerable amount of funds in the treasury; and we are happy to say at this time, that but a very small debt of two or three hundred dollars (over and above the $4,000 which is in the form of a mortgage with four and five years to run) is against the institution. Instead therefore of pressing debts to be paid, the association in another year will in all probability begin to have a fund accumulating in the hands of the treasurer.

It will be the part of wisdom, therefore, to see that the funds which come into his hands be in safe keeping. These funds will accrue from two sources; first the sale of stock which has been authorized; and a small surplus each year in the rent of the college building which is paid by the faculty.

In 1852 it was resolved that the stockholders hereby allow all the moneys accruing from interest on stock, admission of members, and other sources to be appropriated for the next three years for repairs and improvements to the College building. In 1853, the time as above resolved was extended to six years.

Next spring this period will expire, and it will depend on the action of the association, whether this fund be cut off or not.

The stock it will be recollected bears six per cent interest which the faculty pays as a rent, and which is now appropriated to liquidate the debt incurred in the erection of the building. There is also a small fund which yearly comes into the treasury as annual dues from the graduates of the school who may become members of the association, and who are required to pay six per cent on what constitutes a share of stock; we would be glad to see the number of such increased.

As the duty of managing the financial affairs of the institution appears to devolve on the trustees, it would appear proper that the treasurer should belong to that body. I would however recommend that this officer be required to give bond in an amount sufficient to cover any sum which may be placed in his hands, and that for all amounts over and above $100, which may be in his hands for more than three months he shall pay interest at the rate of six per cent. It is hoped that the amount of stock authorized to be sold and other funds paid in, may be sufficient if well husbanded to meet the mortgages on the Institution. The sooner however the stock is sold the more effectually will this be secured; for the stock paid in will be bringing interest to meet that which is paid on the mortgage.

In conclusion, gentlemen, I would remark, that it was not alone the object of our organization to erect this College and take care of its funds, but also for our "mutual improvement and the advancement of Dental science."

When the association has attended to the first object which has so deeply engaged attention, would it not be well to turn attention to subjects of a more directly scientific nature? The stockholders and alumni of the institution, will soon be able to bring together yearly an amount of talent and professional spirit, which shall delight to handle any department of science connected with Dental Surgery in such a manner that our annual convocations shall indeed be "a feast of fat things."

To secure more perfectly the interests of the stockholders, the faculty have increased the amount of policy on the institution to $5,000. It is supposed that this amount would restore the building in case of fire.

Hoping, gentlemen, that the work which this association has so nobly commenced and with such zeal so far consummated, may ever be the pride of the profession, and around which the best talent of the profession will delight to rally,

I submit to your wisdom these suggestions,

JAMES TAYLOR.

On motion Drs. Ullery, Richardson, and Keely, were appointed to fill vacancies in the examining committee.

On motion it was resolved (so as to give time to examine the graduating class) to adjourn to 3 P. M.

SECOND DAY.

Met at 3 P. M. Present: Drs. Taylor, Taft, Chapman, H. R. Smith, Watt, Ullery, Brown and Bonsall.

The minutes of last year were first read, after which the following report was received from the faculty, viz:

*Report of the faculty of the Ohio College of Dental Surgery.*

The faculty of the Ohio College of Dental Surgery respectfully present the following report:

The Infirmary at the close of the session last year, was placed under the charge of Dr. E. Osmond, who was selected from the graduating class according to the rules and regulations contained in our last report. With these regulations we are still well pleased, and we propose to continue them with perhaps slight modification.

Our course of instruction began as announced on the first Monday of November, and continued, without interruption till the present time.

We have also the pleasure of reporting an increase in the size of the class, the number in attendance being twenty-three. We would also refer with pleasure to their courteous demeanor, and their attention to study.

Though the present class is larger than the preceeding one, yet we present fewer graduates. Of our present class of graduates two have attended two full courses in this institution, two have each attended a full course in this, and a course in a regular medical school; one is a practitioner of eight years standing, and has attended one course of lectures in this college.

The Infirmary operations of the session are embraced in the accompanying table:—

| | |
|---|---|
| Teeth filled with gold | 240 |
| " " other material | 100 |
| Teeth extracted | 405 |
| Cases of salivary calculus | 21 |
| Pivot teeth inserted | 11 |
| Nerves destroyed and fangs filled | 20 |
| Full upper sets inserted | 8 |
| " under " " | 6 |
| " Partial | 5 |
| Work repaired (done elsewhere) | 8 |

This list of operations would have been much more extensive had we enjoyed an ordinary season. The coal-famine was disastrous to this department, yet the above list shows that the class was not at a loss for practical work.

GEORGE WATT, DEAN.

On motion of Dr. Ullery it was resolved, that the treasurer of the Dental College Association, be elected annually from the board of trustees and that he shall give bond and security for the safe keeping and proper disbursement of the funds of this association; also that he shall pay interest at the rate of six per cent per annum for all moneys over $100 remaining in his hands over three months.

The society then went into an election for officers for the ensuing year when the following were elected:

*President*—Dr. CHARLES BONSALL.

1*st. Vice-President*—Dr. C. B. CHAPMAN.

2*d. Vice-President*—Dr. J. M. BROWN.

*Secretary*—Dr. J. TAFT.

*Treasurer*—Dr. JAMES TAYLOR.

*Dental Examiners*—Dr. J. S. KING, Pittsburg, Pa., Dr. A. G. STIPHER, Decatur, Ill., Dr. HENRY MUNRO, Lebanon, Ky.

*Medical Examimers*—Dr. CHARLES FISHBACK, Shelbyville, Ind., Dr. SAM. MARTIN, Xenia, O.

Adjourned to meet at 2 P. M. to-morrow.

THIRD DAY.

Met at 2 P. M. Present:—Drs. Spalding, Taylor, Taft, H. R. Smith, J. B. Smith, Watt, Keely, Chapman, Baxter, and Bonsall.

The minutes of the previous meeting were read, and approved.

On motion of Dr. Bonsall, the sum of one thousand dollars was fixed on as the amount of bond to be given by the treasurer for the coming year, it being understood that the amount might be changed from year to year according to circumstances.

The following report was received from the building committee, which was accepted and the committee discharged.

The building committee would make the following report: That to meet the debt of the institution, which according to last year's report, was, up to April 1st 1856, $4.280 58, we first secured $3.000 on or about the 1st of July on a mortgage of five years, interest payable semi-annually at the rate of ten per cent. The remainder 1.280 with the interest which had accrued up to the 1st of July, the committee have had borrowed at twelve per cent per annum, to meet this latter, they have now made arrangements for one thousand dollars at ten per cent per annum, payable semi-annually, from the 20th of this month payable in five years.

The following account current shows the present condition of the treasury:—

| | |
|---|---|
| Amount due April 1st 1856, | $4.280 58 |
| Interest on above to present date aside of mortgage | 273 45 |
| Interest on mortgage of $3000 up to July 1st 1857, | 300 00 |
| Interest " " " 1000 " August 2d 1857, | 50 00 |
| Paid Applegate fees | 6 00 |
| One per cent on 4000 negotiation fee | 40 00 |
| Perfecting title | 5 00 |
| Drawing mortgage | 3 00 |
| Notary's fees on both mortgages | 1 50 |
| Cancelling mortgages | 25 |
| Getting up certificate of stock | 14 00 |
| | $4.973 78 |

| | |
|---|---|
| Cr. | |
| Cash on mortgages | $4000 00 |
| Rent from faculty | 510 00 |
| Stock on McKellops | 106 00 |
| " " Herman | 50 00 |
| " " Webster | 95 40 |
| Samuel Griffith | 13 00 |
| | $4774 40 |
| Showing a balance to be met of | $199 38 |

The committee having thus about consummated the work entrusted to them, and a treasurer now having been elected to take charge of the funds, would beg to be discharged.

CHARLES BONSALL,
JAMES TAYLOR.

Dr. H. R. Smith, professor of Mechanical Dentistry, having notified the association that, hereafter, he would not be able to devote more than five hours per week to the duties of the chair, considerable discussion was had on the subject, when finally, Prof. Smith handed in the following resignation, which, on motion was accepted.

CINCINNATI, February 25, 1857.

To the Trustees of the Ohio College of Dental Surgery, and the members of the Dental College Association.

Gentlemen:—I most respectfully resign my office of Professor of Mechanical Dentistry in the Ohio College of Dental Surgery.

H. R. SMITH.

On motion of Dr. Watt, it was

*Resolved*:—That the thanks of this association be returned to Dr. H. R. Smith for his faithfulness in the discharge of his duties in the Ohio College of Dental Surgery.

On motion adjourned till 2 P. M. to-morrow.

FOURTH DAY.

Met according to adjournment.

Present:—Drs. Taylor, Taft, J. B. Smith, Watt, Keely, Bonsall, Baxter, Ullery and Spalding.

The association went into an election of a candidate to fill the vacancy occasioned by the resignation of Dr. H. R. Smith, which resulted in the recommendation of Dr. Joseph Richardson to the trustees, as a suitable person to fill said vacancy.

On motion, adjourned to meet at this place, at 2 P. M. on Monday, February 15, 1858.

CHARLES BONSALL, *Sec'y.*

